# Distribution, congruence, and hotspots of higher plants in China

**DOI:** 10.1038/srep19080

**Published:** 2016-01-11

**Authors:** Lina Zhao, Jinya Li, Huiyuan Liu, Haining Qin

**Affiliations:** 1 State Key Laboratory of Systematic and Evolutionary Botany, Institute of Botany, Chinese Academy of Sciences, Beijing 100093, P.R. China; 2 University of Chinese Academy of Sciences, Beijing 100049, P.R. China; 3 State Key Laboratory of Urban and Regional Ecology, Research Center for Eco-Environmental Sciences, Chinese Academy of Sciences, Beijing 100085, P.R. China

## Abstract

Identifying biodiversity hotspots has become a central issue in setting up priority protection areas, especially as financial resources for biological diversity conservation are limited. Taking China’s Higher Plants Red List (CHPRL), including Bryophytes, Ferns, Gymnosperms, Angiosperms, as the data source, we analyzed the geographic patterns of species richness, endemism, and endangerment via data processing at a fine grid-scale with an average edge length of 30 km based on three aspects of richness information: species richness, endemic species richness, and threatened species richness. We sought to test the accuracy of hotspots used in identifying conservation priorities with regard to higher plants. Next, we tested the congruence of the three aspects and made a comparison of the similarities and differences between the hotspots described in this paper and those in previous studies. We found that over 90% of threatened species in China are concentrated. While a high spatial congruence is observed among the three measures, there is a low congruence between two different sets of hotspots. Our results suggest that biodiversity information should be considered when identifying biological hotspots. Other factors, such as scales, should be included as well to develop biodiversity conservation plans in accordance with the region’s specific conditions.

Economic development and human activities have caused significant damage to the environment and biodiversity resources. Using limited financial resources to protect species, habitats, and vegetation as much as possible is an important goal of the current efforts in biological diversity conservation, while the primary task in determining “where and which species need to be protected” is to identify priority protection areas. As an important basis for setting up priority protection areas, biodiversity hotspots have become a central issue in numerous problems that need to be addressed^1^,^2^.

Identifying biodiversity hotspots typically requires that species diversity, habitat loss, endemism, and endangered status be considered^3^,^4^. Conservationists have thus proposed the use of the three most commonly utilized maps to show species diversity patterns, namely species richness pattern maps, endangered species pattern maps, and restricted species (endemic species) pattern maps^5^,^6^,^7^. This method relies mainly on species distribution patterns to determine which areas are most concentrated with species distribution, which areas have the most obvious endemism, and which areas have the most endangered species. In addition to methods based on traditional species richness spatial analysis, evolutionary diversity and the evolution of species have also received considerable attention as a means of identifying biodiversity hotspots in recent years due to a focus on long-term information on the evolution of different species^8^,^9^,^10^,^11^,^12^.

Despite this interest, however, analysis of species’ broad-scale spatial distribution patterns based on a localized database and a distribution range geodatabase is the central issue in much macro-ecology and conservation biology research, and traditional methods of determining hotspots based on species spatial distribution patterns have not been abandoned. On the contrary, with more in-depth research on taxa and a comprehensive collection of information, traditional analytical methods have stabilized and have been widely applied in research on numerous important taxa and biogeographic regions^13^,^14^,^15^,^16^,^17^,^18^. Taxa that have been studied in greater depth at a global scale include plants (e.g., vascular plants and bryophytes)^7^,^19^,^20^,^21^ and vertebrates (e.g., mammals, birds, amphibians, and fish); several cross-taxon studies have also been conducted^6^,^7^,^14^,^17^,^22^,^23^,^24^,^25^,^26^,^27^,^28^. Various taxa have been studied at national or regional scales in Asia^29^, Europe^30^,^31^,^32^, Africa^33^,^34^, and other regions. China has also conducted some research in this area, particularly on plants throughout the country. Due to a lack of data, however, many studies have either been limited to a specific region, specific habitat range, or specific taxon, or the scales used were relatively coarse; consequently, these studies do not offer a complete picture of China’s plant diversity^35^,^36^,^37^,^38^,^39^.

In this paper, we analyzed the diversity, endemism, and endangered status of 34,450 kinds of Chinese indigenous higher plants based on three aspects of richness information, namely, species richness (SR), endemic species richness (ER), and threatened species richness (TR), on the most current China’s Higher Plants Red List (CHPRL), which was recently released in 2013 (see Material and Methods). In addition, we compare the biodiversity hotspots analyzed in this paper with hotspots widely examined previously. This comparative discussion, based mainly on the hypothesis that there was a connection between the shape of the species-richness distribution and the identification of richness hotspots^40^, specifically include the following: 1) general species richness patterns of higher plants in China based on using CHPRL data to identify areas with the most concentrated species distribution, areas with the greatest number of endemic species, and areas with the largest number of endangered species; 2) the congruence of the three aspects (SR, ER, TR) and an analysis of the impact of changing the definition of hotspots on this congruence; and 3) a comparison and analysis of the similarities and differences between the hotspots presented in this paper and those employed in previous studies. In order to reduce the impact of using a coarse scale on the assessment results, fine-scale regular hexagon grid cells were used to analyze the geographic coverage of the distribution of all species.

## Results and Analysis

### SR, ER, and TR

**Figure 1:**
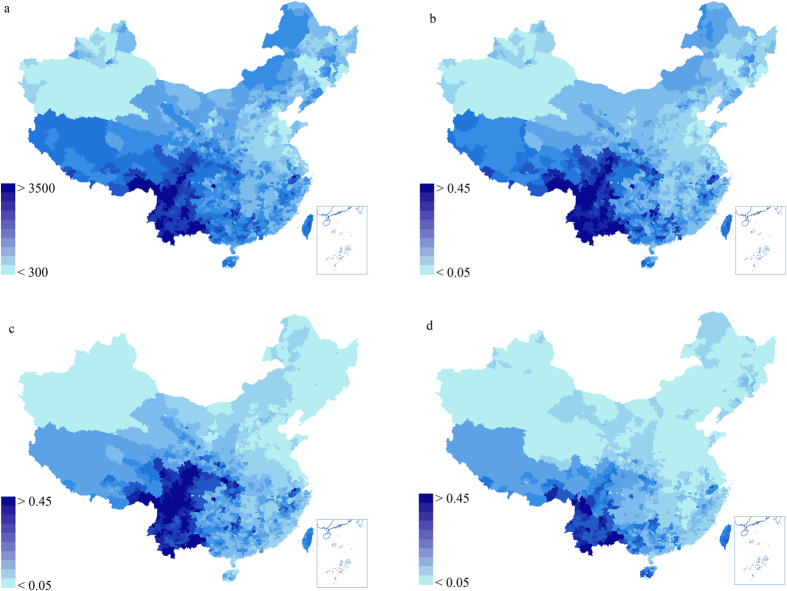
China’s higher plant distribution patterns. (**a**) represents SR; (**b–d**) represent the standardized SR, ER, and TR, respectively. The maps were generated by ArcGIS software (ESRI Inc., Redwoods, USA).

**Figure 2:**
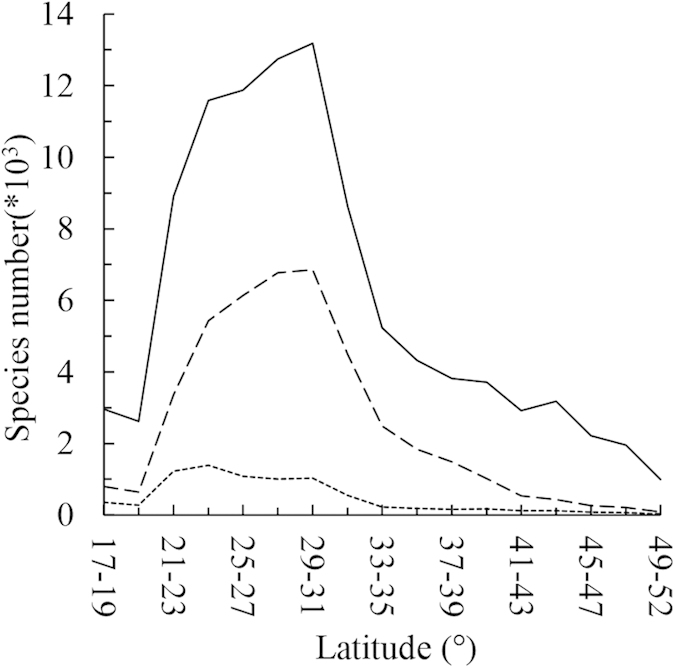
Relationship between latitude and SR (solid line), ER (dash line), and TR (dotted line).

Overall, species richness increases from north to south (Fig. 1), and the entire higher plant richness and endemic species distribution patterns show a unimodal distribution with a peak at latitudes of 29°–31° (Fig. 2), according to which the areas with the most abundant species diversity and the areas with the highest degree of endemism are mainly concentrated in the southwest region. Specifically, these hotspots are located in Yunnan province, Medog County and Zayu County in southern Tibet, the central mountains of Sichuan province, northern Guangxi, the Jinfo Mountains of Chongqing, the Qinling Mountains, the Shennongjia forest district of Hubei province, and the Tianmu Mountains of Zhejiang province, among other places (Fig. 1). In the Southwest, a subtropical evergreen broadleaf forest region, in which the species are most concentrated between latitudes of 21°–35°, contains 89.1% of all species in China and 93.4% of the endemic species, and has the greatest abundance of higher plants in China. Both peak values appear between latitudes of 27°–29° (the junction of Yunnan, Sichuan, and Tibet) (see Figs 1 and 2). In many studies that focus on the number of endemic species, this area is identified as a hotspot for the analysis of the world’s species^2^,^41^.

The threatened species are also concentrated in the Hengduan Mountainous region, Central China, Taiwan, and the Qinling Mountains and its surrounding areas. Figure 1 shows that the distribution patterns of China’s threatened species, endemic species, and species richness have an overall similarity. Figure 2 indicates that threatened species are also concentrated in regions in which species richness and endemic species richness are most abundant; over 90% of threatened species are distributed between latitudes of 21°–35°. Although a high spatial correlation can be found among the three (see Fig. 1, Table 1), there are certain differences in peak values for TR, SR, and ER. Although TR’s distribution seems to resemble a unimodal distribution, it is not as obvious as the distribution of SR and ER, and its peak value does not appear between latitudes of 27°–29°, but rather in latitudes of 23°–25° in the central and southern regions of Yunnan.

### Hotspots definition and congruence

**Figure 3:**
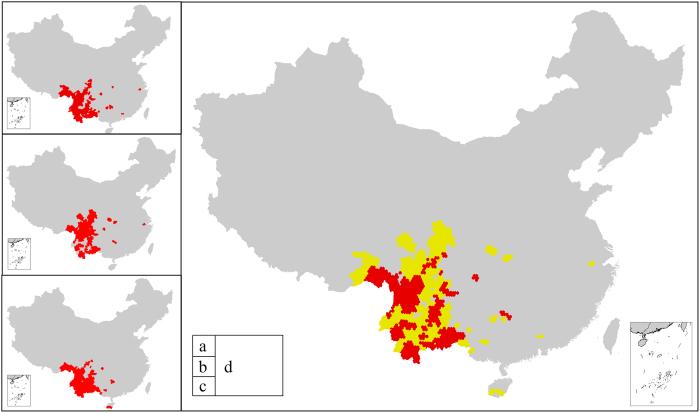
Hotspots, defined as the richest 5% of the cells, obtained based on SR (a), ER (b), and TR (c). Figure (**d**) presents information shared among all three aspects of diversity information (red areas) and information on any of the three aspects of diversity information (red and yellow areas). The maps were generated by ArcGIS software (ESRI Inc., Redwoods, USA).

**Figure 4:**
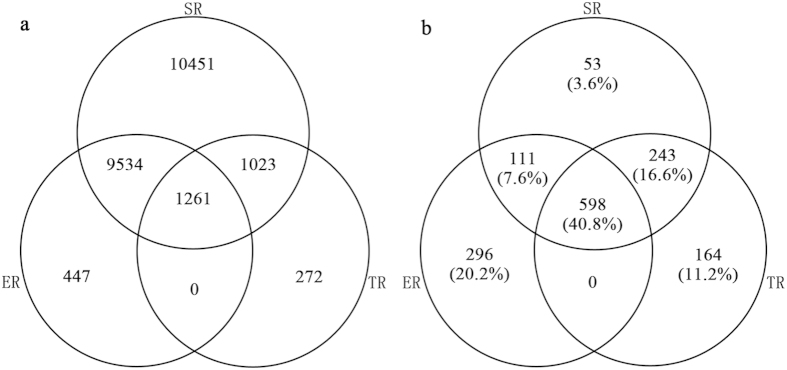
The Venn diagram shows the congruence of SR, ER, and TR (“5%-based hotspots”). The figure on the left shows the congruence in the number of species; the figure on the right shows the congruence in spatial distribution, of which the percentage is its percent of the total hotspots.

Figure 3 shows that species hotspots are mainly concentrated in the mountains of the Southwest, in which species richness is highest under “5%-based hotspots” conditions. We selected the richest 1,005 grid cells for each aspect—SR, ER, and TR. The three aspects accounted for a total of 1,465 grid cells (belonging to at least one of the three aspects, hereafter referred to as the “total hotspots”, depicted in yellow and red in Fig. 3d). Of these, 513 grid cells (35% of the total hotspots) belong to only one of three aspects; 354 grid cells (24.2% of the total hotspots) belong to two aspects; and the remaining 40.8% of the total hotspots are common to all three aspects of diversity, which cover 598 grid cells (Fig. 4), accounting for 3.0% of the total number of grid cells of all flora in China (see the red areas in Fig. 3d). The areas common to all aspects include 1,261 threatened endemic species (accounting for 33.5% of the threatened species, 7.1% of the endemic species, and 3.7% of all species) (Table 2), with most of the areas concentrated near the junction of South Yunnan and Vietnam and the junction of Yunnan, Sichuan, and Tibet.

**Figure 5:**
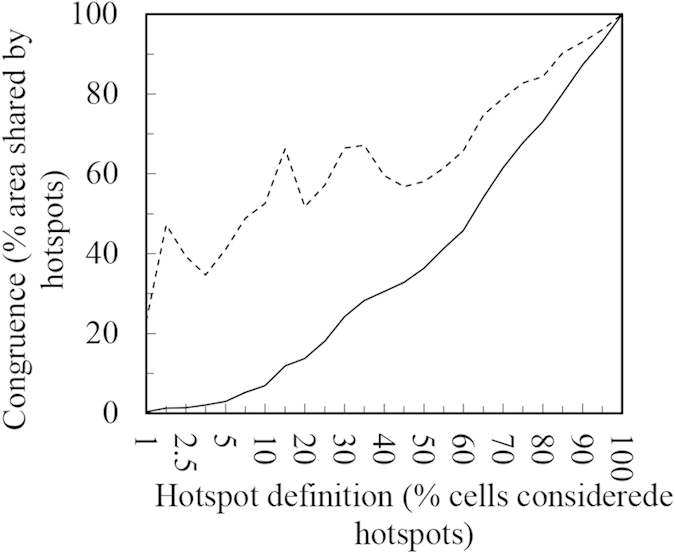
The correlation between the congruence of the three aspects and the hotspots definition. The dashed line shows the ratio of common cells shared by the three aspects to the number of cells taken by the three (the congruence of SR, ER, and TR); the solid line shows the ratio of the area taken by the common cells of SR, ER and TR to the total area of the flora of China.

Spatial congruence is closely related to the manner in which the hotspots were identified^5^. When defining the richest 5% species grid cells as hotspots, the congruence reached 40.8%; the hotpots were shown to contain over one-third of the threatened species (Table 2), even though only 3.0% of the grid cells of all flora were used. Thereafter, we expanded the hotspots definition and analyzed the congruence among SR, ER, and TR. Figure 5 demonstrates that with the expansion of the range of the hotspots definition, aside from occasional instances of reduced congruence, overall congruence among SR, ER, and TR increased, and overall congruence was high. When hotspots were defined as 1%, their congruence was 23.1%; when the definition was expanded to 15%, the congruence approached 66.7%, accounting for 11.9% of the total area of flora and containing over half of the threatened species (51.8%) (Fig. 5).

### Hotspots compared with Myers-criteria-based hotspots

Myers *et al.*^2^ first proposed a list of 25 complete biodiversity hotspots across the globe. The criteria for hotspot designation are mainly based on quantitative indicators of both endemic plant species and habitat degradation: a hotspot must contain at least 1,500 species of endemic vascular plants and must have lost at least 70% of its primary vegetation (hereafter referred to as “Myers-criteria”). Conservation International also selected endemic plant diversity as the main criteria and, based on Myers’ studies, reassessed global biodiversity hotspots to establish the most recent list of 34 biodiversity hotspots (34 hotspots from Hotspots Revisited) (hereafter referred to as “Myers-criteria-based hotspots”)^41^.

**Figure 6:**
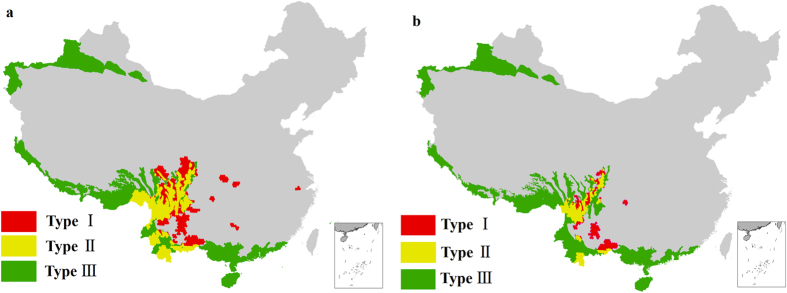
Comparison maps between 5%-based hotspots. (**a**) and 1,500-based hotspots (**b**) and Myers-criteria-based hotspots, of which Type I: belongs to the 5%-based hotspots (or the 1,500-based hotspots) in this paper, but does not belong to the Myers-criteria-based hotspots (red coverage areas); Type II: belongs to both the 5%-based hotspots (or the 1,500-based hotspots) and the Myers-criteria-based hotspots (yellow coverage areas); and Type III: belongs to the Myers-criteria-based hotspots, but does not belong to the 5%-based hotspots (or the 1,500-based hotspots) (green coverage areas). The maps were generated by ArcGIS software (ESRI Inc., Redwoods, USA).

In the process of analyzing the similarities and differences between the hotspots presented in this paper and Myers-criteria-based hotspots, in addition to analyzing the “5%-based hotspots”, we also analyzed the hotspots that meet the Myers-based criteria, according to which “the number of endemic species is no less than 1,500” (hereafter referred to as “1,500-based hotspots”), and compared these hotspots with 5%-based hotspots (see Fig. 6). The yellow areas can be classified both as hotspots assessed in this paper (“5%-based hotspots” in Fig. 6a and “1,500-based hotspots” in Fig. 6b) and as “Myers-criteria-based hotspots.” Green areas cannot be categorized as either of the hotspots assessed in this paper, but are considered to be Myers-criteria-based hotspots. The red areas represent those hotspots analyzed in this paper that do not meet the requirements for being categorized as Myers-criteria-based hotspots. These areas should be of great concern, as these areas may belong to priority conservation areas, but have not yet received the attention they deserve. The results of the comparison demonstrate that “Myers-criteria-based hotspots” and the hotspots obtained based on the two criteria in this paper do not completely overlap (see Table 3, Fig. 6). Compared with the 5%-based hotspots, only 53.4% of the areas overlap, just over half the coverage of the hotspots. Even if the hotspots are obtained using the same “Myers criteria” (“1500-based hotspots”), the completely overlapping area only amounts to 11,2961 km^2^, accounting for 56.2% of the total area of the hotspots assessed in this paper, and only 9.5% of the total area of “Myers-criteria-based hotspots” in China (Fig. 6b). Overall, 43.8% of the area of the hotspots assessed in this paper, approximately 87,889 km^2^, was listed as hotspots (see the red areas in Fig. 6b). This area, which does not fall into the “Myers-criteria-based hotspots” range, should nevertheless be included in priority conservation areas.

## Discussion

### Patterns and potential determinants of species in China

The results show that the distribution patterns of China’s species richness and endemic species richness are roughly the same^42^ (Fig. 1), which is concordant with the findings of previous studies^42^,^43^. Although exceedingly rich in species, China’s higher plant species richness and endemic species richness are neither uniformly distributed nor randomly distributed; they do, however, follow certain rules. China’s species richness patterns exhibited an overall trend of more species being distributed in the South and less species being distributed in the North, with distribution mainly concentrated in the mountainous southwest region of China. Relevant research results from this paper also confirmed this point^37^. Geological history theory generally believes that richness, nature, and community aspects of mountainous Southwest China and their geographic distribution patterns are mainly controlled by changes in the land and sea environments and formations of mountains and plateaus, as well as by the complex and diversified eco-environment^42^. In terms of climate, Yunnan and Sichuan are located in the second step, which is affected by both southern subtropical monsoons and East Asian monsoons, giving rise to a humid and diversified climate. In addition, the uplift and orogeny of the Tibetan Plateau has resulted in high topographic heterogeneity and has established a variety of complex regional ecological environments, resulting in extremely rich species diversity, while also providing sufficient conditions to produce the unusually high degree of local endemic species^44^. Since species richness and endemic species richness are affected by nearly the same factors, they have a high congruence in terms of spatial distribution (R = 0.93, p < 0.01).

The spatial distribution of endangered species and risk factors are closely related. The local people have damaged a large area of forests to reclaim the land for production and living^26^. Many species are extremely vulnerable to becoming endangered or even to going extinct due to habitat loss, which can be a reason for the difference between the peak value of endangered species and that of all species and endemic species (Fig. 2). Overall, however, the patterns of endangered species have a high congruence with those of all species and of endemic species (R ≥ 0.83), as species habitats in regions with a high degree of endemic species are fragile, and most endemic species belong to species with a restricted range, which are susceptible to outside interference, with endemic species extremely vulnerable to becoming endangered^2^,^11^. Consequently, the distribution patterns of the three different aspects of species are roughly the same, as each of them have a unimodal distribution. However, the TR has a slightly different peak. The determinants may be that, in addition to the association with species distribution itself, the distribution patterns of endangered species are closely correlated with human activities, including studies of the taxa itself. When analyzing taxa congruence, the object of study, which may be less well-known taxa, a gap in the study area, or species data, may not have been completely collected, may also affect the analysis of congruence. The same holds true for hotspots.

### Congruence and spatial scales influence

Much attention has been paid to the study of biodiversity hotspots as it provides ways to assess protection priorities^3^,^9^,^11^,^45^. With regard to China’s entire flora, SR, ER, and TR are highly correlated with one another (Table 1), and the hotspots obtained based on the analysis of the species biodiversity information offered by the three aspects also have a high congruence in spatial distribution (Table 2, Fig. 3). The high diversity congruence is of great significance for species conservation. Species diversity, the degree of endemism, and threatened status and other biodiversity information are often used as a basis for considering the protection of particular species^1^,^41^,^45^. However, it should be noted that species diversity rose in accordance with a fluctuation trend, not always adhering to the rule that the larger the percentage, the higher the congruence. The congruence of SR, ER, and TR shown in Fig. 5 indicate that species diversity rose while adhering to a fluctuation trend and that these aspects have several extrema values, which may be helpful in guiding conservation policy. When the hotspots for each of these three aspects are relatively concentrated, important species can be protected to the greatest extent possible using fewer resources.

However, hotspots of species richness, endemism, and threat were not always congruent in other taxa. For example, Orme *et al.* (2005) found that hotspots of species richness, endemism, and threat do not show the same geographical distribution and found a surprisingly low overall congruence of the three biodiversity measures when using a new global database of all known extant bird species. Only 2.5% of hotspot areas are common to all three aspects of diversity when the richest 2.5% of grid cells with respect to species richness, threat, or endemism, respectively were used. And the level of congruence has been shown not to be merely an artifact of the initial hotspot definition in both the study conducted by Orme *et al.* (2005) and this paper. But the overall changing trend in the congruence is on the rise, which agrees with our findings. In addition, Ceballos *et al.*(2006) found low congruence among hotspots based on richness, restricted-range species, and threatened species when they tested the efficiency of hotspots for conservation of mammalian diversity and the congruence among hotspots (only 1% of cells were common to all three types under the 2.5% criterion,). These cases also show the necessity of congruence analysis with comparison between hotspots.

The conclusion that there is a high congruence among the three types of hotspots in China’s higher plants is conducive to improving efficiency and centralized management when allocating limited resources to protecting species, habitats, and vegetation as much as possible in China. Congruence among hotspot types also has implications for the use of hotspots in conservation. If congruence were, in fact, high, then each index of diversity would be suitable for guiding conservation policy because any one of such indices could act as an effective surrogate for the other aspects of diversity. If not, multiple indices of diversity would be needed to identify areas of high conservation priority.

Congruence between the 5%-based hotspots of higher plants in this paper and the Myers-criteria-based hotspots is low. Even when applying Myers criteria that hotspots have “no less than 1,500 endemic plant species,” a large difference in coverage of the hotpots is obtained. Possible reasons include, the following, among a multitude of other reasons: 1) Myers criteria take vascular plant endemism into account but have no rigorous quantitative criteria for non-vascular plants; and 2) Myers criteria are closely correlated with habitat degradation, but the degree of habitat degradation in different vegetation taxa (species habitat) varies and cannot be defined by a uniform standard. In addition, habitat degradation processes are not repeatable^4^.

In addition to the reasons listed above, spatial grain is an important issue for congruence in many ecological studies. In the global scale biodiversity pattern study, cells with a side length of 1° or even larger scales are usually used as basic cells of data analysis^5^,^7^,^17^,^26^,^46^, but this practice is not suitable for local areas with large spatial heterogeneity and extremely rich biodiversity^40^. Nevertheless, in many study areas in which a fine scale is applied in studying the spatial distribution of taxa, due to a lack of data, researchers have relied exclusively on hotspots data and have avoided non-hotspots data that has not been researched as extensively, giving rise to a certain tendency in analysis results and thereby affecting assessment results 15.

## Conclusion

This paper identified many important but ignored hotspots based on an analysis of species richness, endemic species richness, and endangered species richness. These hotspots are rich in terms of species with a high degree of endemism and a very serious endangered status quo, which should be the focus of future biodiversity protection efforts. Naturally, it is recommended that many other informative layers be analyzed in addition to richness and patterns to identify hotspot range and conservation priority areas, including layers with multi-taxa distribution or habitat information, or other layers that may prove valuable. Due to resource constraints, conservation efforts to help all endemic or threatened species cannot be implemented at present^47^. What can be done is to use the least and best resources to protect species as much as possible, particularly those most in need of protection. Therefore, this paper suggested that aside from focusing on identifying hotspots in reference to global scales, an appropriate grid size in line with its own region be applied in analyzing biodiversity phenomena in order to formulate a biodiversity conservation action plan aligned with the needs and specific circumstances of the region.

## Material and Methods

### Species data and geographic data

The species data stem mainly from the CHPRL released in September 2013^48^. The list presents the latest information on species provided by nearly 300 experts and includes all the extinction risk levels of all known higher plants in China based on IUCN Red List Categories and Criteria (v. 3.1)^49^. The list covers nearly all known wild higher plants native to China (totaling 34,450 species, including subspecies, deadline 2013), including 2,494 kinds of Bryophytes, accounting for 7.2% of all higher plants; 2,177 kinds of Ferns, accounting for 6.7% of all higher plants; 249 kinds of Gymnosperms, accounting for 0.7% of all higher plants; and 29,530 kinds of Angiosperms, accounting for 85.7% of all higher plants^48^. Threatened species identified in the IUCN Red List are categorized into the following three categories: Critically Endangered (CR), Endangered (EN), and Vulnerable (VU) species, totaling 3,767 species, which could be downloaded at http://www.mep.gov.cn/. China’s endemic higher plants amount to 17,700 species, accounting for 51.4% of all higher plants in China; these endemic species have great value in terms of germplasm resources and genetic diversity. The assessment results reveal that among China’s endemic species of higher plants, 2,462 are threatened species, accounting for 13.9% of the total endemic species (17,700) and 65.4% of the total threatened species (3,767) (see Table 4).

The species distribution data was essential information for our analysis. In addition to collecting geographic data from nearly 300 Red List program experts, for certain species and main provincial flora, we also made reference to *Flora Republicae Popularis Sinicae*, *Flora of China,* and *Higher Plants of China*, as well as a large number of journal articles (see Appendix S1 for supporting information). In addition, for detailed geographic information on certain species, we referred to information on approximately 1.2 million unique distributions of Herbarium specimens presented by CVH (Chinese Virtual Herbarium, http://www.cvh.org.cn/cms/en/, accessed Dec 2014), NSII (National Specimen Information Infrastructure, http://www.nsii.org.cn/, accessed Dec 2014), and other large specimen platforms. The latest version of these data sources were selected and were used together to enrich the database. As for the location of a given species, the data with specific and more accurate geographical information would be selected based on the China’s administrative region division (2007).

## Methods

Species richness (SR) is defined as the number of species distributed in each grid cell. The term endemic species encompasses species whose distribution is only restricted to a particular region, while endemic species richness (ER) refers to the number of endemic species in each grid cell. Meanwhile, the term threatened species refers to the number of threatened species in each grid cell. All species diversity information stems mainly from the latest CHPRL. As the degree of complexity among taxa differs and as there is a huge quantity variance among taxa, large taxa often dominate the distribution patterns of biodiversity in the analysis, while smaller taxa are often in a subordinate position. In order to balance these differences between taxa and reduce the dominance of large taxa, we standardized the diversity of species. We definded a biodiversity index as the ratio of the actual number of species in a particular grid cell to the largest number of species in all grid cells^17^, which is expressed as follows:


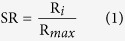


where *R*_i_ is the value of SR in the *i-*th cell, while *R*_max_ is the largest value of SR in all cells. For example, if the greatest richness is 1000 and there are 300 species in a cell, the cell is assigned a 0.3. The treatment for ER and TR is the same as that used for SR.

When analyzing the spatial distribution of species, we used geographic distribution data (Extent of Occurrence of IUCN Red List, EOO) for a comprehensive collection of species distribution information based on the existing Red List database and other databases. To differ from studies based on a “localized database”, the method based on the “distribution range” (EOO) used to conduct species diversity analyses mainly considers species distribution range and potential distribution range, while studies based on a “localized database” mainly take a particular distribution range set as the object of analysis. After overlapping all the species range maps using Geographic Information System technology^17^, we gridded overlapped layers into hexagon cells of the same size, with values equal to the number of species. In the selection of grid size, a side length of 1° grid has been used in numerous studies^5^,^7^,^13^,^22^; however, a smaller grid size is available for analysis of the distribution patterns of species. In this paper, a grid size 20 times finer was used; that is to say, a side length of 1° grid was divided into 20 small hexagon grid cells that are commonly used in biodiversity studies, thereby dividing the entire flora of China into approximately 20,000 valid grid cells in the form of regular hexagons with an average edge length of 30 km.

We used grid-processed maps to analyze species diversity, hotspots, and spatial congruence, and then used generalized linear mixed models (GLMM) to analyze the spatial correlation of diversity information on the three different aspects. In analysis of the congruence, we defined the richest 5% biodiversity area as hotspots (hereafter referred to as “5%-based hotspots”)^50^. We then identified SR, ER, and TR hotspots, respectively and analyzed the spatial distribution congruence between each pair. All geographic information was processed using ArcGIS software (ESRI Inc., Redwoods, USA).

## Additional Information

**How to cite this article**: Zhao, L. *et al.* Distribution, congruence, and hotspots of higher plants in China. *Sci. Rep.* **6**, 19080; doi: 10.1038/srep19080 (2016).

## Supplementary Material

Supplementary Appendix S1

## Figures and Tables

**Table 1:** The correlation between SR, ER and TR with the definition of 5%-based hotspots (p < 0.001).

Pearson’s r	SR	ER	TR
SR	1		
ER	0.93	1	
TR	0.92	0.83	1

**Table 2:** Congruence analysis results of SR, ER and TR with the definition of 5%-based hotspots.

5%-based hotspots	Cells			Species		
	Cells No.	% of total cells common to all three aspects	% of total cells of Chinese plant flora	Species No.	% of total species in the three aspects	% total species in SR^1^ or ER^2^ or TR^3^
SR	1005	69.0	5.0	22269	93.5	64.7
ER	1005	69.0	5.0	11242	47.2	63.6
TR	1005	69.0	5.0	2556	10.7	67.9
Common to all three aspects	598	40.8	3.0	1261	5.3	33.5
Total in all hotspots	1465	100	7.2	23812	100	–

SR^1^: 34398 species (excluding 52 species assessed as Extinct); ER^2^: 17664 endemic species (not including endemic species assessed as Extinct); TR^3^: 3767 species (Threatened categories) (see Material and Methods).

**Table 3:** Geographic area and the percentage of different aspects of hotspots.

	5%-based hotspots				1500-based hotspots				Myers Hotspots
	Area (km^2^) or Number of species	%^1^	%^2^	%^3^	Area (km^2^) or Species No.	%^1^	%^2^	%^3^	
Total area	540002	100		5.6	200850	100		2.1	1190983
Type I	288431	53.4	24.2	3.0	112961	56.2	9.5	1.2	–
Type II	251571	46.6	21.1	2.6	87889	43.8	7.4	0.9	–
Type III	902552	–	75.8	9.4	902551	–	75.8	9.4	–

%^1^: The area of three Types (I, II, III) compared with that of 5%-based hotspots or that of 1500-based hotspots.

%^2^: The area of three Types (I, II, III) compared with that of Myers-criteria-based hotspots.

%^3^: The area of three Types (I, II, III) compared with that of the entire flora of China.

**Table 4:** Categories and Percentage of CHPRL.

Categories	Total no. species (% of all higher plants)	Total no. endemic species (% of all the species in each category)
Extinct (EX)	27(0.1)	27(100)
Extinct in wild (EW)	10(0.03)	9(90)
Regional Extinct (RE)	15(0.04)	0(0)
Critically Endangered (CR)	583(1.7)	456(78.2)
Endangered (EN)	1297(3.8)	851(65.6)
Vulnerable (VU)	1887(5.5)	1155(61.2)
Near Threatened (NT)	2723(7.9)	1771(65)
Least Concern (LC)	24296(70.5)	11098(45.7)
Data Deficient (DD)	3612(10.5)	2333(64.6)
Total	34450	17700(51.4)
