# Supplementary material for: Distribution, congruence, and hotspots of higher plants in China
Source: Sci Rep. 2016 Jan 11;6:19080. doi: 10.1038/srep19080 (PMC4707485; doi:10.1038/srep19080)
Supplement: Supplementary Appendix S1 [file srep19080-s1.doc]

**Distribution, congruence, and hotspots of higher plants in China**

Lina Zhao^1,†^, Jinya Li^2,†^, Huiyuan Liu^1^, Haining Qin^1,*^

**Appendix** S1. Literature list of supplemental information of geographical distribution in the analysis.

1. Chen H (1964) Flora of Hainan. Science Press, Beijing.
2. Chen H (1965) Flora of Hainan. Science Press, Beijing.
3. Chen H (1990) Flora of Shandong. Qingdao Press, Qingdao.
4. Chen H, Zheng Y, Li F (1997) Flora of Shandong. Qingdao Press, Qingdao.
5. Chen Q (2004) Flora of Guizhou. Guizhou Science and Technology Press, Guiyang.
6. Committee of science and technology of Fujian (1982-1995) Flora of Fujian. Fujian Science and Technology Press, Fuzhou.
7. Ding B, Wang S (1981) Flora of Henan. Henan People’s Press, Zhengzhou.
8. Ding B, Wang S (1988) Flora of Henan. Henan Science and Technology Press, Zhengzhou.
9. Ding B, Wang S (1997) Flora of Henan. Henan Science and Technology Press, Zhengzhou.
10. Ding B, Wang S, Gao Z (1998) Flora of Henan. Henan Science and Technology Press, Zhengzhou.
11. Editorial Committee of Flora Reipublicae Popularis Sinicae (1959-2004) Flora Reipublicae Popularis Sinicae. Science Press, Beijing.
12. Editorial Committee of Higher Plants of China (1999-2005) Higher plants of China. Qingdao Press.
13. Editorial Committee of Flora Flora of Anhui (1986) Flora of Anhui. Anhui Science and Technology Press, Hefei.
14. Editorial Committee of Flora Flora of Anhui (1987) Flora of Anhui. China Prospective Press, Beijing.
15. Editorial Committee of Flora Flora of Anhui (1990) Flora of Anhui. China Prospective Press, Beijing.
16. Editorial Committee of Flora Flora of Anhui (1991) Flora of Anhui. China Prospective Press, Beijing.
17. Editorial Committee of Flora Flora of Anhui (1992) Flora of Anhui. Anhui Science and Technology Press, Hefei.
18. Editorial Committee of Flora Flora of Guizhou (1982) Flora of Guizhou. Guizhou People Press, Guiyang.
19. Editorial Committee of Flora Flora of Guizhou (1986a) Flora of Guizhou. Guizhou People Press, Guiyang.
20. Editorial Committee of Flora Flora of Guizhou (1986b) Flora of Guizhou. Guizhou People Press, Guiyang.
21. Editorial Committee of Flora Flora of Guizhou (1988) Flora of Guizhou. Sichuan National Press, Chengdu.
22. Editorial Committee of Flora Flora of Guizhou (1989a) Flora of Guizhou. Sichuan National Press, Chengdu.
23. Editorial Committee of Flora Flora of Guizhou (1989b) Flora of Guizhou. Sichuan National Press, Chengdu.
24. Editorial Committee of Flora Flora of Guizhou (1989c) Flora of Guizhou. Sichuan National Press, Chengdu.
25. Editorial Committee of Flora Flora of Guizhou (1989d) Flora of Guizhou. Sichuan National Press, Chengdu.
26. Editorial Committee of Flora Flora of Hebei (1986-1991) Flora of Hebei. Hebei Science and Technology Press, Shijiazhuang.
27. Editorial Committee of Flora Flora of Jiangsu (1982) Flora of Jiangsu. Jiangsu Science and Technology Press, Nanjing.
28. Editorial Committee of Flora Flora of Jiangsu (1997) Flora of Jiangsu. Jiangsu People Press, Nanjing.
29. Guangdong Institute of Botany, the Chinese Academy of Sciences (1974) Flora of Hainan. Science Press, Beijing.
30. Guangdong Institute of Botany, the Chinese Academy of Sciences (1977) Flora of Hainan. Science Press, Beijing.
31. Kunming Institute of Botany, the Chinese Academy of Sciences (1977-2005) Flora Yunnanica. Science Press, Beijing.
32. Wu C (1983-1987) Flora of Xizang. Science Press, Beijing.
33. Wu Z, Raven PH, Hong D (1994-2006) Flora of China. Science Press (Beijing) & Missouri Botanical Garden Press (St. Louis).
